# *APOE4* allele in north Indian elderly patients with dementia or late onset depression-a multiple-disease case control study

**DOI:** 10.22099/mbrc.2019.34417.1427

**Published:** 2019-09

**Authors:** Anamika Misra, Sankha Shubhra Chakrabarti, Indrajeet Singh Gambhir, Upinder Kaur, Shukla Prasad

**Affiliations:** 1 Department of Geriatric Medicine, Institute of Medical Sciences, Banaras Hindu University, Varanasi, India; 2 Department of Pharmacology, Institute of Medical Sciences, Banaras Hindu University, Varanasi, India; 3 Department of Zoology, Institute of Science, Banaras Hindu University, Varanasi, India

**Keywords:** Alzheimer’s disease, APOE, Depression, Vascular dementia

## Abstract

The objective of the study was to analyze the frequency of *APOE4* allele in elderly patients with Alzheimer’s or vascular dementia or depression; compare these to age/sex matched controls; compare the results with established literature and highlight new findings. A single center, multiple disease, case-control study was performed with three case groups- probable AD patients (n=36), vascular dementia patients (n=29) and depression patients (n=20) and with a control group (n=32). *APOE *genotyping was performed in whole blood samples collected from patients and controls by restriction isotyping using the enzymes AflIII and HaeII. There was significant difference in frequency distribution of *E4* allele between the AD (12/72; 16.7%) and control groups (3/64; 4.7%) (P=0.03). However, no significant difference was found in any of the other comparisons. The current study demonstrates absence of a significant association between *APOE4* positivity and presence of late-onset depression in the north Indian elderly and reinforces the higher *APOE4* prevalence in LOAD patients but not in VD patients. It is the first study of its kind from the northern part of India involving multiple disease groups and lays the framework for larger cohort studies.

## INTRODUCTION

Age-related neurological diseases are becoming a major concern as world populations grow older. Dementia and depression account for a large proportion of age-related neurological diseases [1]. Alzheimer’s disease (AD) is the most common form of dementia and is followed by vascular dementia in prevalence [2]. Apolipoprotein E gene (*APOE*) is the major risk gene identified as yet for late onset AD (LOAD), the commonest form of AD, which has an onset at more than 60 years of age [3, 4] The APOE protein serves as a ligand for low density lipoprotein receptor, and participates in the transport of cholesterol and other lipids among various cells [5]. APOE protein is encoded by a gene mapped on chromosome 19 and has three major isoforms: E2, E3, E4 [6]. Greater accumulation of both amyloid- beta plaques and neurofibrillary tangles, the two pathologic hallmarks of AD is associated with the presence of E4 allele while the E2 allele may confer protection against AD [7].

The association of *APOE4* with AD has been demonstrated in the north Indian population. It is similar to that seen in western data but prevalence of *APOE4* allele is comparatively low as compared to other allelic forms [8]. The *APOE4* allele has been also found to be significantly associated with dementia among south Indian patients, with *APOE4* allele frequencies being higher among AD patients (0.27) as compared to controls (0.08) [9]. However, the link between *APOE* and other forms of dementia such as vascular dementia is still inconclusive, more so in the Indian population [10]. An alteration in cholesterol metabolism caused by the *APOE4 *allele may be expected to affect central nervous system vascular diseases such as vascular dementia [11]. 

According to the World Health Organization, depression was the fourth leading contributor to the global burden of disease in society in the year 2000 [12]. Depression is common in patients with AD [13]. Late onset depression has also been considered to increase the risk of AD, but its association with *APOE * genotype status is not clear [14]. A novel study from south India suggested that *APOE4* allele is associated with late onset depression (LOD) and it is reported that elderly with an *APOE4* allele are more prone to develop depression in old age [15]. Such findings again have not been demonstrated in the ethnically distinct north Indian elderly population. 

With these findings in consideration, the current study aimed to estimate the relative frequency of *APOE4* allele in north Indian elderly patients with LOAD, vascular dementia or depression; compare these to the *APOE4 *allele frequency in age/sex matched controls; and interpret the findings thereof.

## MATERIALS AND METHODS

**Study design: **The current study had a single center, multiple disease (group), case-control design with three case groups- probable AD patients, vascular dementia patients and depression patients and with an age/gender matched control group.

**Biological sample collection, processing and storage: **2 mL of non-fasting blood samples were collected from all the subjects under study in sterile EDTA anticoagulant vials and were stored in 4^0^C. All collected samples were processed within 24 hours to isolate DNA by phenol chloroform method [16]. Extracted DNA from each sample was stored in high quality Eppendorf tubes under refrigeration at -20^0^C. 

**Study setting and participants: **The study was carried out at the geriatric out-patients’ services of a large tertiary care teaching hospital in north India from June 2013 to May 2016. Patients ≥60 years of age, suspected to have dementia or depression based on initial history were evaluated and enrolled for the study by random selection, after obtaining written informed consent. The patients of probable AD (n=36) and vascular dementia (n=29) were diagnosed using the DSM-5 criteria after initial screening with the Hindi Mental State Examination (HMSE; cutoff score for dementia-≤23) [17, 18]. The diagnosis was supported by magnetic resonance imaging of brain which showed characteristic pattern of cerebral atrophy seen in AD as determined by an expert radiologist, while ruling out other causes such as cerebrovascular disease; and showed significant vascular lesions (multi-infarct state or old large infarct/ bleed) in cases of vascular dementia. Depression patients (n=20) were clinically diagnosed using the DSM-5 criteria after initial screening with the Geriatric Depression Score- short form (cutoff score for depression:≥5) [19]. The healthy controls (n=32), age and sex matched, were randomly chosen from the community. All healthy controls underwent neuropsychological evaluation to exclude dementia, and informed consent was taken from each, to include them in the study. For AD and vascular dementia patients, the informed consent was taken either from the patient or the primary caregiver of the patient in cases of advanced disease. The study was approved by the institutional ethics committee.

Patients with any severe acute illness requiring hospitalization or leading to delirium, depression diagnosis or onset before 60 years of age, and any condition such as severe deafness, blindness or other illness which would interfere with neuropsychological testing were excluded from the study. In the depression group, only those patients without co-existent dementia were included.

**Study parameters: **The basic demographic data of each participant of the study, including HMSE & GDS scores were collected in pre-designed case report form. For all the subjects under this study, *APOE *genotyping was performed and the determined types were entered into the case report form. *APOE *genotyping was performed as per standard protocol in literature [20].

**Statistical Methods:** Descriptive statistics were used to express demographic data. One-way ANOVA was used in comparing the groups for baseline quantitative variables such as age, HMSE score and GDS score. Gender distribution was compared between the groups using Chi-square test. The statistical difference in the distribution of allelic variants in controls versus cases was calculated using Fisher's exact test or χ^2^ test as appropriate. A value of *P*<0.05 was considered statistically significant. The statistical analysis was performed by using SPSS version 16 (SPSS Inc, Chicago, USA). 

## RESULTS

The male: female ratio and age did not differ statistically between the four groups (Table 1). The Hindi Mental State Examination (HMSE) score and Geriatric Depression Scale (GDS- short form) score in the four groups are depicted in Table 2. As expected, HMSE score were significantly lower in dementia patients (AD&VD) as compared to controls or depression patients. Further, the Geriatric Depression Scale (short form) score was significantly higher in the depression group as compared to each of the other three groups.

**Table 1:** Demographic profile of the study groups

	**Alzheimer’s Disease N=36**	**Vascular Dementia N=29**	**Depression N=20**	**Control N=32**
**Age (yrs) (Mean** **± SD)**	71.6 ± 9.6	68.5 ± 7.8	66.7 ± 6.8	67.2 ± 6.5
**Gender (Male/ Female)**	27/9	18/11	10/10	26/6

**Table 2:** Hindi Mental State Examination (HMSE) score and Geriatric Depression Score (GDS) in the groups

	**Alzheimer’s Disease**	**Vascular Dementia**	**Depression**	**Control**
**HMSE (mean **± SD)*	18.1 ± 3.6	18.6 ± 3.7	28.3 ± 1.9	28.9 ± 1.4
**GDS (mean **±** SD)**^#^	3.4 ± 2.5	2.9 ± 2.7	9.8 ± 2.5	1.8 ± 1.7

*p value <.001 for comparison between AD patients and controls, AD patients and depression patients, VD patients and controls and VD patients and depression patients (insignificant for other comparisons) ^#^p value <.001 for comparison between depression patients and controls, depression patients and AD patients, depression patients and VD patients (insignificant for other comparisons) [Independent t-test and Mann-Whitney U test as per normality]

Genotypes of patients/ controls in the study population is represented in Table 3. There was significant difference in frequency of E4 allele between the AD (12/72;16.7%) and control groups (3/64; 4.7%) (*P*=0.03). The frequency of *APOE4 *allele in the vascular dementia group was 12.5% (5/40) and in the depression group was 12.1% (7/58). However, no significant difference was found in distribution of E4 allele in any of the other comparisons i.e. AD *vs* VD (*P*=0.46); AD *vs* depression (*P*=0.56); depression *vs* control (*P*=0.25); VD *v*s control (*P*=0.19); and VD *vs* depression (*P*=0.95).

**Table 3:** Distribution of *APOE* genotypes between the study groups

**Study Group (N=117)**	**E3/E4**	**E3/E3**	**E2/E3**	**E2/E4**
**Alzheimer’s Disease (N= 36)**	33.3% (12/36)	63.9% (23/36)	2.8% (1/36)	0
**Vascular Dementia (N=29)**	20.7% (6/29)	72.4% (21/29)	3.4% (1/29)	3.4% (1/29)
**Depression (N=20)**	25% (5/20)	70% (14/20)	5% (1/20)	0
**Control (N=32)**	9.4% (3/32)	87.5% (28/32)	3.1% (1/32)	0
**TOTAL**	22.2% (26/117)	73.5% (86/117)	3.4% (4/117)	0.9% (1/117)

## DISCUSSION

*APOE4* is the prime genetic risk factor for LOAD. However, the relation between *APOE4* and late onset depression which may be a precursor of LOAD, a feature of LOAD or an independent morbidity in the elderly has been inconsistent across world literature. Literature search revealed only one major article from India [15]. This particular study however included a predominant south Indian population who are ethnically and phenotypically quite different from the north Indian population. Similarly, the relationship between *APOE4 *genotype and vascular dementia has been scarcely explored in the north Indian population. The current study is the first effort of its kind in the north Indian elderly in exploring *APOE4* gene frequency in multiple patient groups- AD, VD and late onset depression with respect to healthy controls.

The association of *APOE4* allele with LOAD is highly conserved among different populations, and elevated frequency of E4 in AD patients versus controls has been observed in multiple ethnic groups [21]. Compiled data from 40 research teams showed that the association of the E4 allele with AD was strongest in the Japanese population, followed by Caucasians and lastly the African-Americans and Hispanics [21]. In our study, there was greater prevalence of the E4 allele in the AD group with respect to the control group which is in line with world literature.

The association between E4 allele and VD has been inconsistent and a matter of controversy [22]. Data suggests that APOE4 elevates the risk of VD, but not to the same extent as of AD [22]. In the current study, no significant difference was found in distribution of *E4* allele in between VD and control groups. Larger studies may be warranted to validate the results.

It has been shown in an European population that the E4 allele occurs in late-onset depression with a frequency similar to that in AD [23]. Another European study involving 42 patients with AD, 26 early-onset depression (EOD) patients, 23 late-onset depression patients and 49 controls also showed that the frequency of E4 allele in LOD but not EOD, was similar to that in AD and higher than that in controls [24]. In the single Indian study of this type, the odds of individuals with an E4 allele of developing depression in old age was 4.7 times that of those without an E4 allele [15]. However, a study from the Czech Republic demonstrated an increased probability of comorbid depression in AD patients who do not carry the E4 allele [25]. This seems in conflict with the other findings. The pathophysiologic basis of how *APOE4* may result in late-life depression is unclear as yet. In our study, no significant difference was found in *E4* allele distribution between the patients of depression and the control group but the frequency of E4 allele was found to be 12.1% in depression patients which was apparently high as compared to control group (4.7%). A larger sample size may result in altered conclusions. The patients of depression enrolled did not have dementia at the time of selection but in the absence of follow-up, it could not be ensured that some of them would not develop dementia in future. Late onset depression has been considered to be a precursor of both AD and vascular dementia [26]. This may also have a bearing on the observed frequencies of *E4 *allele. 

The current study is the first one to explore the relationship between *APOE4* and varied categories of neuropsychiatric disorders in the elderly in the north Indian population. The only previous study from south India involved only two study groups, namely depression patients and healthy controls. In fact, the authors mentioned the absence of other control groups such as AD patients as one of the shortcomings. Keeping this in mind, we tried to involve multiple disease comparison groups, making the findings more robust. However, absence of serial follow-up and a small sample size remain significant short comings of the study. The enrollment of patients using random selection instead of including all consecutive patients may also be considered a limitation of the study though, random selection was computerized and free from investigator bias. 

The current study demonstrates absence of a significant association between *APOE4* positivity and presence of late-onset depression in the north Indian elderly and also reinforces the widely known concepts that *APOE4* prevalence is higher in LOAD patients but not in VD patients. The most salient highlight of the study is that it opens up the possibility that late-onset depression may not always be a precursor of LOAD and there may be distinct groups within these patients. While *APOE4* may be more prevalent in the group which would proceed to LOAD; the other group of late-onset depression patients may have *APOE4* prevalence similar to healthy controls. Efforts may be focused on discovering clinical patterns and other biomarkers particular to those late-onset depression patients, who may progress to LOAD. Whether *APOE4* plays a role in determining this may be analyzed in cohort studies.
